# microRNA-1298 inhibits the malignant behaviors of breast cancer cells via targeting ADAM9

**DOI:** 10.1042/BSR20201215

**Published:** 2020-12-10

**Authors:** Weili Chen, Qing Lu, Siyu Li, Xinyue Zhang, Xiaohong Xue

**Affiliations:** Department of Mammary, YueYang Hospital of Integrated Traditional Chinese and Western Medicine, Shanghai University of Traditional Chinese Medicine, Hongkou District, Shanghai City, China, 200080

**Keywords:** ADAM9, Breast cancer, miR-1298

## Abstract

MicroRNAs (miRNAs) regulate the progression of human malignancy by targeting oncogenes or tumor suppressors, which are 12 promising targets for cancer treatment. Increasing evidence has suggested the aberrant expression and tumor-suppressive function of miR-1298 in cancers, however, the regulatory mechanism of miR-1298 in breast cancer (BC) remains unclear. Here, our findings showed that miR-1298 was down-regulated in BC tissues and cell lines. Lower level of miR-1298 was significantly correlated with the advanced progression of BC patients. Experimental study showed that overexpression of miR-1298 inhibited the proliferation, induced apoptosis and cell cycle arrest in BC cells. The *in vivo* xenograft mice model showed that highly expressed miR-1298 significantly reduced the tumor growth and metastasis. Further mechanism analysis revealed that miR-1298 bound the 3′-untranslated region (UTR) of a disintegrin and metalloproteinase 9 domain (ADAM9) and suppressed the expression of ADAM9 in BC cells. ADAM9 was overexpressed in BC tissues and inversely correlated with miR-1298. Down-regulation of ADAM9 induced apoptosis and cell cycle arrest of BC cells. Moreover, ectopic expression of ADAM9 by transiently transfecting with vector encoding the full coding sequence of ADAM9 attenuated the inhibitory effects of miR-1298 on the proliferation and cell cycle progression of BC cells. Collectively, our results illustrated that miR-1298 played a suppressive role in regulating the phenotype of BC cells through directly repressing ADAM9, suggesting the potential application of miR-1298 in the therapy of BC.

## Introduction

Breast cancer (BC) is one of the most common malignancies and the second leading cause of tumor-related deaths among women worldwide [1,2]. Although the application of chemotherapy, radiotherapy and surgical resection has improved the survival rate of BC patients over the past decade, the prognosis of BC patients is still not unsatisfactory. The high incidence of drug resistance results into treatment failure and death in patients experiencing distant metastasis. Therefore, it is urgent to identify new diagnostic markers and therapeutic targets to improve the outcome of patients with BC.

Accumulating evidence has demonstrated the potential roles of microRNAs (miRNAs) in the diagnosis and treatment of cancers [3–7]. miRNAs are characterized as a class of single-stranded, small non-protein coding RNAs with the length of ∼22 nucleotides [8,9]. miRNAs regulate the gene expression via binding to the 3′-untranslated region (UTR) of target mRNAs [10]. The critical function of miRNAs in multiple biological processes, such as cell proliferation, invasion and metastasis, has been reported [11]. Interestingly, increasing evidence shows that miRNAs are important regulators in BC progression, which serve as potential diagnostic and prognostic biomarkers [12,13]. For example, miR-137 is found to suppress the development of triple-negative BC via targeting Del-1 [14]. miR-33a-5p sensitizes BC cells to doxorubicin via inhibiting eIF5A2 expression and the process of epithelial–mesenchymal transition [15]. The plasma level of miR-21 is significantly increased in primary and recurrent BC patients, which maybe a useful biomarker for the detection of BC [16]. Notably, recent studies show the down-regulation of miR-1298 in several human cancers, which is associated with the lymph node metastasis and TNM stage of cancer patients [17–20]. Overexpression of miR-1298 suppresses the proliferation and invasion of bladder cancer, non-small cell lung cancer and gastric cancer [18–20]. These findings indicate miR-1298 as a specific promising target for the therapy of cancer patients. However, the function of miR-1298 in BC remains largely unknown.

The aim of the present study is to detect the expression of miR-1298 in BC tissues and characterize the role of miR-1298 in the malignant behaviors of BC cells. The targets of miR-1298 were also identified and their roles in BC were also studied.

## Materials and methods

### Clinical specimens

A total of 50 paired BC tissues and adjacent normal tissues were collected from patients (age range, 29–74 years; female) who underwent surgical resection at the YueYang Hospital of Integrated Traditional Chinese and Western Medicine between August 2011 and December 2013. Tissues were immediately frozen in liquid nitrogen and stored at −80°C before further experiments. The present study was performed in accordance with the Helsinki Declaration and was approved by the Ethics Committee of the YueYang Hospital of Integrated Traditional Chinese and Western Medicine. Written informed consents were obtained from all the patients enrolled in the present study.

### Cell culture and transfection

The BC cell lines including MCF-7, MDA-MB-231, BT-20, SKBR-3 and normal human mammary epithelial cell line MCF-10A were obtained from the American Type Culture Collection (ATCC; Manassas, VA, U.S.A.). Cells were cultured in DMEM containing 10% fetal bovine serum (FBS, Invitrogen; Thermo Fisher Scientific, Inc. Waltham, MA, U.S.A.) at 37°C with 5% CO_2_.

Cells were transfected with the 50 nM of miR-1298 mimic, mimic negative control (NC-miRNA) or miR-1298 inhibitor, inhibitor-NC using Lipofectamine 2000 reagent (Invitrogen; Thermo Fisher Scientific, Inc.) according to the manufacturer’s instructions. Cells were harvested after transfection for 48 h for the following experiments.

### Reverse transcription-quantitative PCR

Total RNA was extracted from the tissues or cell lines using the TRIzol reagent (Takaro Bio, Inc.) according to the manufacturer’s instructions. The reverse transcription of cDNA was performed with the PrimeScript RT Reagent Kit (Thermo Fisher Scientific, Inc.) according to the guidelines. The relative expression of miR-1298 and a disintegrin and metalloproteinase 9 domain (ADAM9) was evaluated via the qPCR using the SYBR Green PCR Mix (Bio-Rad, U.S.A.) on the LightCycler System (Roche Diagnostics GmbH). The thermocycling conditions for qPCR were as follows: 95°C for 5 min followed by 40 cycles of 95°C for 15 s and 55°C for 45 s. Expression levels of U6 RNA and GAPDH were detected as the internal control of miR-1298 and ADAM9 and normalized using the 2^−ΔΔ*C*_T_^ method.

### Cell counting kit-8 assay

MCF-7 and MDA-MB-231 cells transfected with miR-1298 mimic or miR-NC were seeded into the 96-well plate with 1000 cells per well. After culturing at 37°C for 1-, 2-, 3-, 4- and 5 days, 10 μl of cell counting kit-8 (CCK-8) reagent was added into the medium and incubated for additional 4 h at 37°C. The absorbance of each well was determined at 450 nm with the microplate spectrophotometer (Elx800; BioTek Instruments, Inc., Winooski, VT, U.S.A.). The experiment was performed in triplicates.

### Western blot analysis

Total proteins were extracted using the RIPA lysis buffer (Beyotime, Shanghai, China) containing protease inhibitor (Solarbio, Beijing, China). The protein concentration was determined with the Bradford Protein Assay (Bio-Rad Laboratories, Inc.) according to the manufacturer’s instructions. An equal amount of protein (20 μg) was loaded into each well of the 15% SDS/PAGE and transferred on to the PVDF membranes (EMD Millipore, Billerica, MA, U.S.A.). The membrane was blocked with 5% non-fat milk for 1 h at room temperature (RT) followed by incubating with primary antibody against ADAM9 (1:1000; ab186833, Abcam, Shanghai, China) or GAPDH (1:2000; ab181602, Abcam, Shanghai, China) overnight at 4°C. After washing twice with TBST (0.1% Tween-20), the membrane was incubated with horseradish peroxidase-conjugated secondary antibody at RT for 1 h. The protein blots were detected using the enhanced chemiluminescence solution (EMD Millipore, Billerica, MA, U.S.A.) with the ChemDoc Imaging System (Bio-Rad Laboratories, Inc., Hercules, CA, U.S.A.).

### Luciferase reporter assay

The wildtype (WT) or mutant (Mut) 3′-UTR oligonucleotides of ADAM9 containing the putative binding sites of miR-1298 were cloned into the pMIR-REPORT luciferase-expressing vector (EMD Millipore, Billerica, MA, U.S.A.). The constructs were co-transfected with miR-1298 mimic or miR-NC into MCF-7 or MDA-MB-231 cells using the Lipofectamine 2000 (Invitrogen; Thermo Fisher Scientific, Inc.). After transfection for 48 h, cells were harvested and the luciferase activity was determined using the Dual-Luciferase Reporter Assay Kit (Promega Corporation, Madison, WI, U.S.A.) according to the manufacturer’s instructions. The experiment was performed in triplicates.

### Cell apoptosis and cell cycle analysis

The apoptosis of BC cells was analyzed using the Dead Cell Apoptosis Kit with Annexin V Alexa Fluor™ 488 and Propidium Iodide (V13241, Thermo Fisher Scientific, U.S.A.) according to the manufacturer’s instructions. For the cell cycle analysis, cells were fixed overnight with 70% ice-cold ethanol at 4°C. After washing twice with PBS, cells were incubated with 1 mg/ml RNase A and 50 mg/ml PI for 30 min at RT. The cell cycle distribution was detected using the Cell Lab Quanta SC flow cytometry (Beckman Coulter, Inc.) and the cell cycle prolife was analyzed using the ModFit software (LT, V3.3, Beckman Coulter, Inc.).

### *In vivo* mice assay

The animal experiment was performed at the Experimental Animal Center of YueYang Hospital and approved by the Ethics Committee of Yue Yang Hospital of Integrated Traditional Chinese and Western Medicine. The nude mice (female, BALB/c; 4–5 weeks; 15–20 g) were purchased from the Charles River Laboratories, China (Beijing, China) and housed at RT under a 12-h light/dark cycle with free access to water and food. BC cells were infected with the lentivirus vector of miR-1298 mimics or miR-NC, respectively. Cells (1 × 10^6^) were subcutaneously injected into the flanks of nude mice. Tumor growth was monitored and mice were killed after 30 days via cervical dislocation. Tumor volume (V) was calculated as 0.5 × length × width^2^.

### Biotin-based pull-down assay

The biotin-based miR-1298 pull-down assay was performed as previously described [21]. Briefly, biotin-labeled miR-1298 or miR-NC was synthesized and transfected into the BC cells. Cells were lysed and the lysate was incubated with magnetic streptavidin beads. RNA was extracted from the beads and cDNA synthesis was performed. The binding between ADAM9 and miR-1298 was detected by qPCR using the primers targeting the open reading frame (ORF) region or the 3′-UTR of ADAM9.

### Statistical analysis

The statistical analysis was performed using GraphPad Prism software (Version 6.0, GraphPad Software Inc.). The differences between groups were determined using the Student’s *t* test and multiple groups were performed using the one-way analysis of variance (ANOVA) followed by Tukey’s post hoc test. The correlation between the expressions of miR-1298 and ADAM9 in BC tissues was tested with the Spearman correlation test. *P*<0.05 was considered as statistical significance.

## Results

### miR-1298 was down-regulated in BC tissues and cell lines

**Figure 1:**
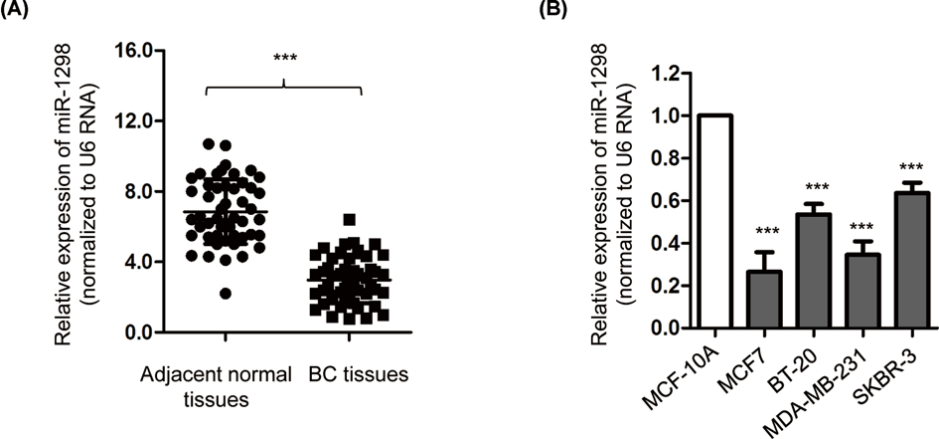
miR-1298 was down-regulated in BC tissues and cells (**A**) The expression levels of miR-1298 in BC tissues were significantly decreased compared with the adjacent normal tissues (*n*=50). (**B**) The level of miR-1298 was frequently down-regulated in BC cell lines in comparison with that of the normal MCF-10A cells. ***P<0.001.

To evaluate the potential involvement of miR-1298 in BC, the expression of miR-1298 in BC tissues and paired adjacent normal tissues was determined by reverse transcription-quantitative PCR (RT-qPCR). The result showed that the expression of miR-1298 was significantly lower in BC tissues than that of the adjacent normal tissues (Figure 1A). Furthermore, as presented in Figure 1B, the level of miR-1298 was also obviously decreased in BC cell lines compared with that of the normal mammary epithelial MCF-10A cells. To evaluate the clinical significance of miR-1298, those 50 patients were divided into miR-1298-low or miR-1298-high groups according to the mean expression value of miR-1298. The correlation between the level of miR-1298 and the clinical characteristics of BC patients was analyzed. As indicated in Table 1, low level of miR-1298 was significantly correlated with the tumor size, TNM stage, high histological grade and lymph node metastasis of BC patients (Table 1). These results suggested the down-regulation of miR-1298 may be involved in the progression of BC.

**Table 1:** The correlation between the expression of miR-1298 and the clinical features of BC patients

Clinical characteristics	Case	High-miR-1298	Low-miR-1298	*P*-value
**Age, years**				
≤60	24	9	15	*P*>0.05
>60	26	9	17	
**Tumor size (cm)**				
≤4	23	11	12	*P*<0.01
>4	27	7	20	
**Histological grade**				
Low	19	13	6	*P*<0.001
High	31	5	26	
**Lymph node metastasis**				
Negative	18	10	8	*P*<0.001
Positive	32	8	24	
**TNM stage**				
I–II	18	8	10	*P*<0.001
III–IV	32	10	22	

### miR-1298 inhibited the proliferation, induced apoptosis and cell cycle arrest of BC cells

**Figure 2:**
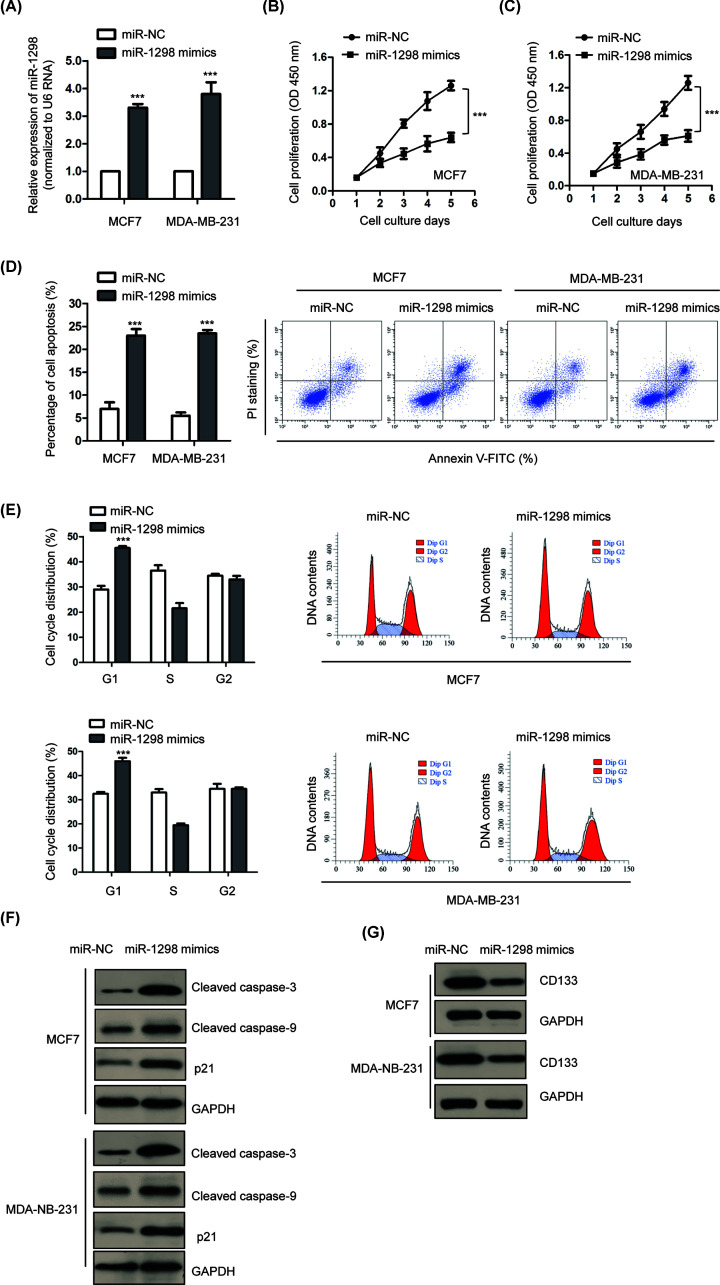
Overexpression of miR-1298 inhibited the growth of BC cells (**A**) RT-qPCR was performed to evaluate the transfection efficiency of miR-1298 mimic in BC cells. (**B,C**) Overexpression of miR-1298 inhibited the proliferation of MCF7 and MDA-MB-231 cells. (**D**) The apoptosis of BC cells was significantly increased with the transfection of miR-1298. (**E**) Compared with control cells, overexpression of miR-1298 in BC cells induced cell cycle arrest at G_1_ phase. (**F**) Overexpression of miR-1298 induced the cleavage of caspase 3/9 and accumulation of p21. (**G**) miR-1298 reduced the expression level of CD133 in BC cells. ***P<0.001.

MCF7 and MDA-MB-231 cells were transfected with miR-1298 mimic or miR-NC because of the lower level of miR-1298 among all the BC cell lines we used in Figure 1B. The overexpression of miR-1298 was confirmed by RT-qPCR analysis (Figure 2A). To investigate the effects of miR-1298 on the growth of BC cells, CCK-8 assay was performed with BC cells expressing miR-1298 mimic or miR-NC. The results showed that overexpression of miR-1298 significantly inhibited the proliferation of both MCF7 and MDA-MB-231 cells (Figure 2B,C). The influence of miR-1298 on the growth of BC cells was also investigated by determining the cell apoptosis. Flow cytometry analysis revealed that overexpression of miR-1298 significantly increased the apoptosis of both MCF7 and MDA-MB-231 cells compared with that of cells carrying miR-NC (Figure 2D). Additionally, the cell cycle progression of BC cells expressing miR-1298 mimic or miR-NC was also determined by the flow cytometry. As indicated in Figure 2E, overexpression of miR-1298 obviously increased the percentage of cells that distributed in G_1_ phase and decreased in S phase, suggesting G_1_ cell cycle arrest with highly expressed miR-1298. To support the conclusion that miR-1298 induced apoptosis and cell cycle arrest of BC cells, the expression of apoptosis-related cleaved caspase-3/9 and the accumulation of p21 that acts in G_1_ to S phase transition was detected. The results showed that overexpressed miR-1298 increased the cleavage of caspase-3 and 9, as well as the abundance of p21 in BC cells (Figure 2F), which was consistent with the promoted apoptosis and cell cycle arrest of BC cells. Moreover, to investigate the possible effects of miR-1298 on the differentiation of BC, the expression of CD133 that is associated with the cell self-renewal and differentiation was evaluated. As indicated in Figure 2G, overexpression of miR-1298 reduced the levels of CD133 in both MCF7 and MDA-MB-231 cells, suggesting the negative possible role of miR-1298 in the differentiation of BC. Collectively, these results demonstrated that miR-1298 might play an inhibitory role in the malignancy of BC.

### Overexpressed miR-1298 inhibited the tumor growth *in vivo*

**Figure 3:**
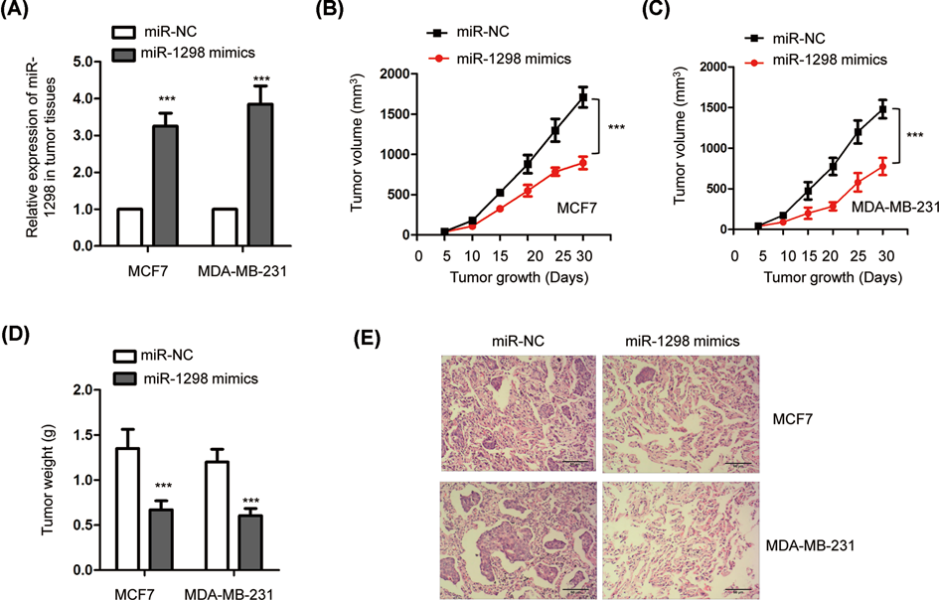
miR-1298 inhibited the tumor growth *in vivo* (**A**) The level of miR-1298 in tumor tissues was detected by RT-qPCR. (**B,C**) The tumor volume was significantly decreased with highly expressed miR-1298. (**D**) Overexpression of miR-1298 reduced the tumor weight in nude mice. (**E**) Highly expressed miR-1298 in tumors reduced incidence to lung metastasis.

To validate the suppressive role of miR-1298 in BC, *in vivo* xenograft mice model was established. MCF7 or MDA-MB-231 cells infected with lentivirus vector that stably expressing miR-1298 mimics or miR-NC were subcutaneously injected into the flank of nude mice. Mice were killed via cervical dislocation after 30 days. The overexpression of miR-1298 in tumor tissues expressing from each group was validated by RT-qPCR (Figure 3A). The tumor volume and weight was compared between the experimental and control groups after 30 days. As presented in Figure 3B,C, overexpression of miR-1298 in both MCF7 and MDA-MB-231 cells significantly suppressed the tumor volume (Figure 3B,C) compared with the control group. Similarly, the tumor weight was also obviously reduced with the highly expressed miR-1298 (Figure 3D). Furthermore, the xenograft tumors from control cells expressing miR-NC developed metastases to the lung, while cells harboring miR-1298 mimics showed reduced incidence of metastasis (Figure 3E). These results indicated that miR-1298 inhibited the tumor growth *in vivo*.

### ADAM9 was a direct target of miR-1298

**Figure 4:**
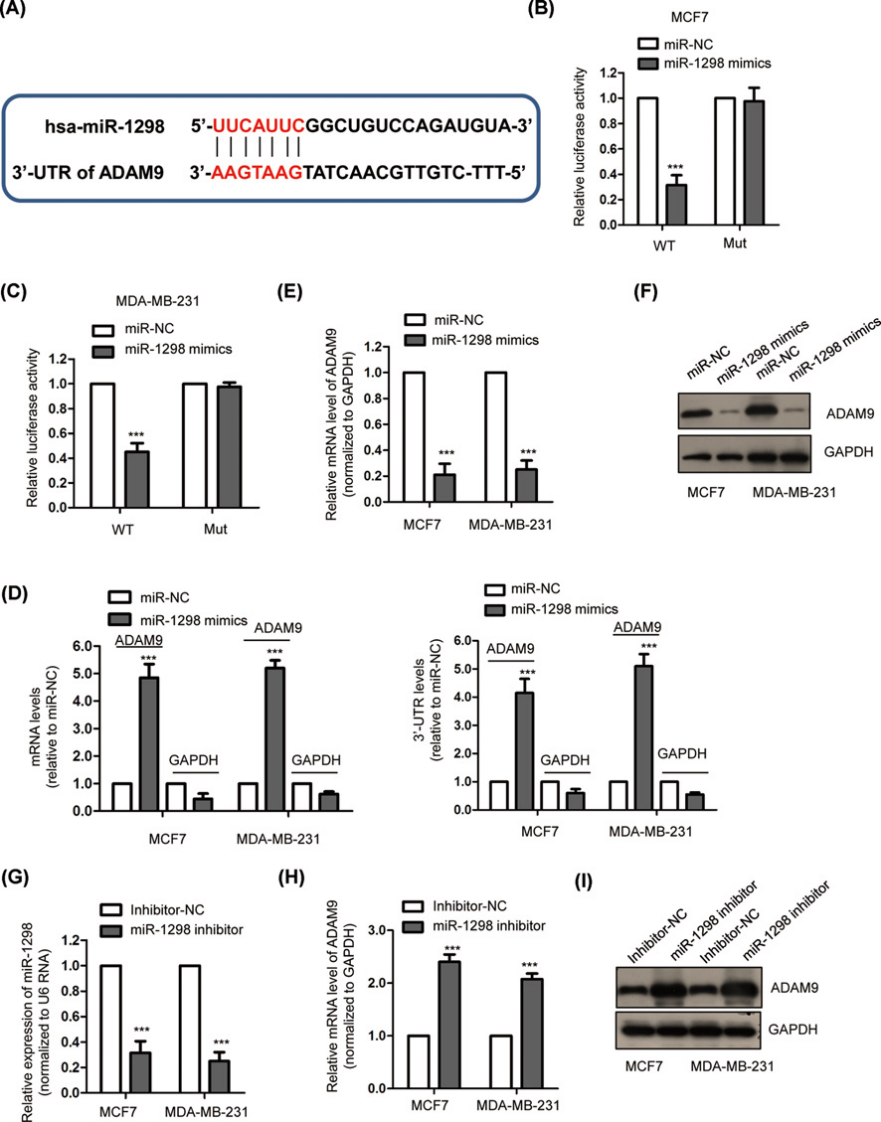
ADAM9 was a target of miR-1298 (**A**) The predicted miR-1298 binding sites in the 3′-UTR of ADAM9 mRNA. (**B,C**) Dual-luciferase reporter assay was performed following co-transfection with WT or MUT luciferase report vector and miR-1298 mimic or miR-NC. miR-1298 overexpression resulted into reduced activity of WT but not the Mut reporter vector. (**D**) The identification of ADAM9 as a target of miR-1298 by qPCR. Left panel, the mRNA levels which amplified the ORF region of targeted mRNAs. Higher level of ADAM9 was observed compared with GAPDH (negative control). Right panel, amplification of the 3′-UTR of ADAM9 or GAPDH. (**E,F**) Overexpression of miR-1298 significantly decreased the mRNA and protein levels of ADAM9 in both MCF7 and MDA-MB-231 cells. (**G**) BC cells were transfected with miR-1298 inhibitor or inhibitor-NC, and the knockdown efficiency of miR-1298 was confirmed by RT-qPCR. (**H,I**) Transfection of miR-1298 inhibitor in both MCF7 and MDA-MB-231 cells markedly increased the expression of ADAM9. ***P<0.001.

To further understand the role of miR-1298 in BC, the potential targets of miR-1298 were predicted using the miRDB online target prediction tool. The putative complementary binding sties of miR-1298 were found within the 3′-UTR of ADAM9 (Figure 4A). To confirm this prediction, luciferase reporter assay was performed by co-transfecting miR-1298 and luciferase vector harboring WT or Mut 3′-UTR of ADAM9. The results showed that overexpression of miR-1298 significantly inhibited the luciferase activity of WT-3′-UTR of ADAM9 (Figure 4B,C). However, the luciferase activity of Mut-3′-UTR of ADAM9 was not affected with the transfection of miR-1298 (Figure 4B,C). To provide more evidence for the binding between miR-1298 and the 3′-UTR of ADAM9, we also performed biotin-based pull-down assay to validate the mRNA targets [21]. Our results showed that amplification of the 3′-UTR of ADAM9 or the ORF region of ADAM9 was significantly enriched by biotinylated miR-1298. No amplification was observed for the negative control GAPDH (Figure 4D). These results demonstrated the binding between miR-1298 and the 3′-UTR of ADAM9.

To investigate whether the binding of miR-1298 affected the mRNA abundance of ADAM9, the expression of ADAM9 in BC cells with the transfection of miR-1298 mimic or mimic-NC was detected by RT-qPCR assay. The data indicated that overexpression of miR-1298 resulted into a significant reduction in ADAM9 mRNA expression (Figure 4E). Consistently, decreased protein level of ADAM9 was also observed with the overexpression of miR-1298 in both MCF-7 and MDA-MB-231 cells (Figure 4F). To further evaluate the influence of miR-1298 on the expression of ADAM9, the level of miR-1298 was down-regulated by introducing miR-1298 inhibitor into BC cells (Figure 4G). Results demonstrated that knockdown of miR-1298 significantly increased both the mRNA and protein levels of ADAM9 in BC cells compared with cells carrying inhibitor control (Figure 4H,I). Collectively, these results indicated ADAM9 was targeted and negatively regulated by miR-1298 in BC cells.

### ADAM9 was overexpressed in BC and inversely correlated with miR-1298

**Figure 5:**
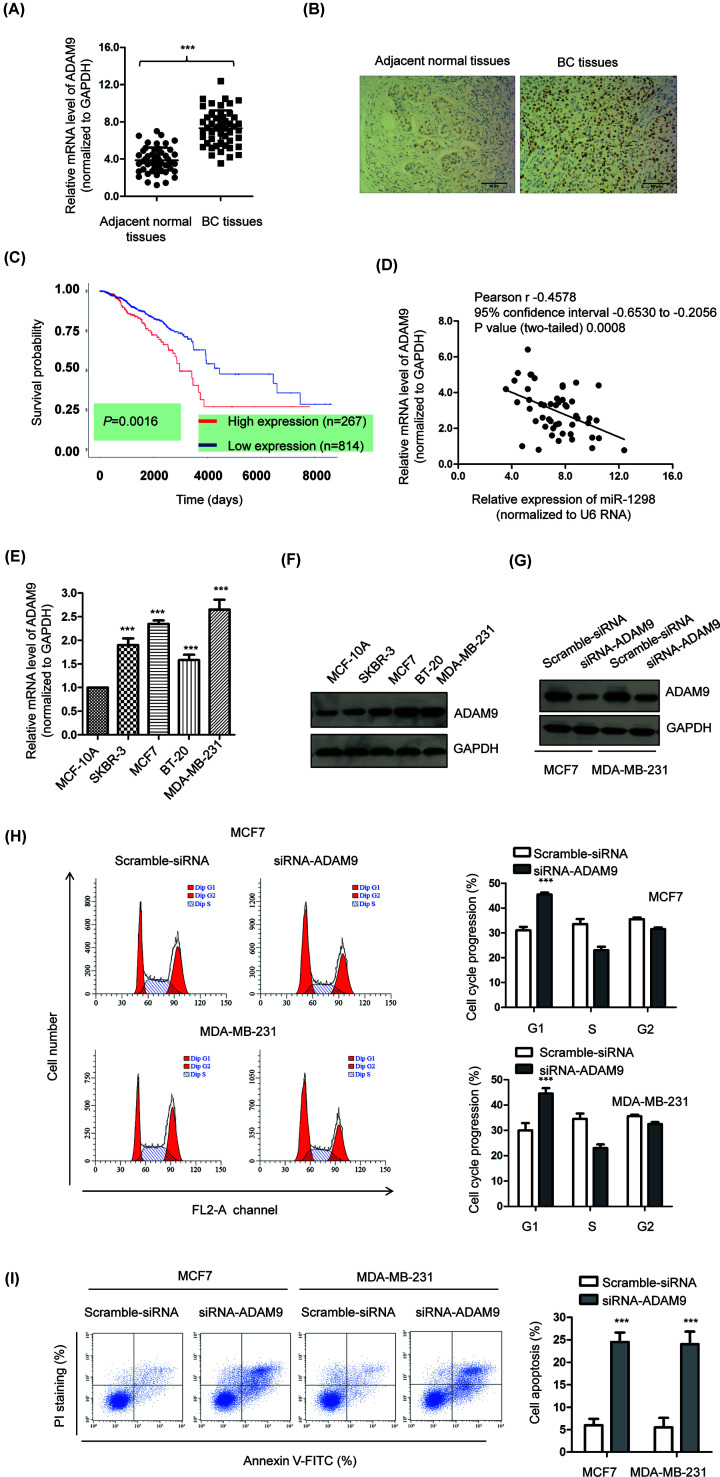
ADAM9 was highly expressed in BC and inversely correlated with the expression of miR-1298 (**A**) Expression levels of ADAM9 were determined in 50 paired BC tissues and corresponding adjacent non-cancerous tissues using RT-qPCR. (**B**) The IHC staining of ADAM9 in BC tissues and adjacent normal tissues. Scale bar, 50 μm. (**C**) The correlation between the expression of ADAM9 and survival probability of BC patients predicted using the TCGA database. (**D**) Spearman correlation test was performed to determine the relationship between miR-1298 and the mRNA level of ADAM9. (**E,F**) Both the mRNA and protein levels of ADAM9 were higher in BC cell lines than that of the normal MCF-10A cells. (**G**) The down-regulation of ADAM9 was confirmed by western blot. (**H,I**) Knockdown of ADAM9 induced cell apoptosis and cell cycle arrest in BC cells.

To explore the role of ADAM9 in BC, the expression of ADAM9 in BC tissues and paired adjacent normal tissues was examined by RT-qPCR analysis. The abundance of ADAM9 was significantly increased in BC tissues compared with that of the non-cancerous tissues (Figure 5A). The aberrant expression of ADAM9 in BC was also confirmed by immunohistochemical staining with BC tissues and non-cancerous tissues. Higher staining intensity of ADAM9 was observed in BC tissues than that of the adjacent normal tissue (Figure 5B). To evaluate the clinical significance of ADAM9 in BC, the correlation between the expression of ADAM9 and survival probability of BC patients was predicted using the TCGA database. The data showed that the prognosis of BC patients harboring higher level of ADAM9 was worse than those carrying lower expression of ADAM9 (Figure 5C). Moreover, since ADAM9 was identified as a target of miR-1298, the correlation between the expression of miR-1298 and ADAM9 in BC tissues was also evaluated by Spearman correlation test. As indicated in Figure 5D, the level of miR-1298 was negatively correlated with that of ADAM9 in those 50 BC tissues we collected. Additionally, the expression of ADAM9 in BC cells and normal MCF-10A cells was also compared. The results showed that both the mRNA and protein abundance of ADAM9 was frequently higher than that of the control normal cells (Figure 5E,F). To evaluate the effects of ADAM9 on the growth of BC cells, ADAM9 was down-regulated by transfecting siRNA-ADAM9 into both MCF7 and MDA-MB-231 cells. The knockdown efficiency was examined by Western blot (Figure 5G). The cell cycle progression of cells expressing siRNA-control or siRNA-ADAM9 was determined by the flow cytometry. The result showed that down-regulation of ADAM9 significantly induced cell cycle arrest at G_1_ phase (Figure 5H). Furthermore, the apoptosis of both MCF7 and MDA-MB-231 cells was also obviously increased with the down-regulation of ADAM9 (Figure 5I). Therefore, these results demonstrated the highly expressed ADAM9 in BC and down-regulation of ADAM9 inhibited the growth of BC cells.

### Restoration of ADAM9 reversed the inhibitory effects of miR-1298 on the growth of BC cells

**Figure 6:**
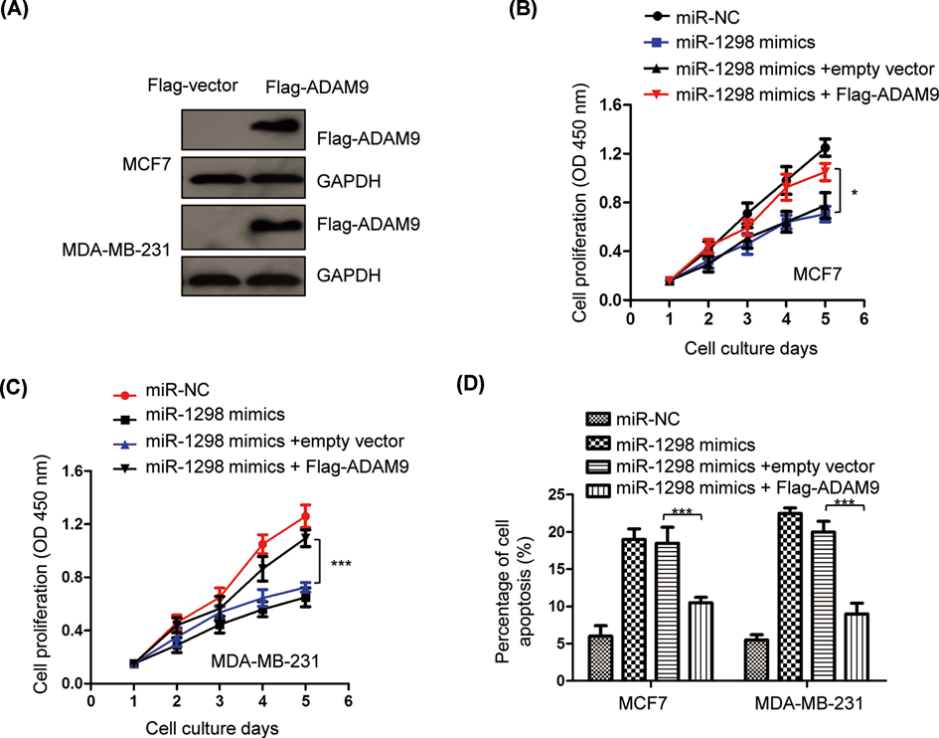
ADAM9 overexpression reversed the anti-cancer effects of miR-1298 on the proliferation and apoptosis of BC cells (**A**) The transfection efficiency of Flag-tagged ADAM9 in BC cells was detected by Western blot using anti-Flag antibody. (**B,C**) Reintroduction of ADAM9 significantly inversed the inhibitory effects of miR-1298 on the proliferation of MCF7 and MDA-MB-231 cells. (**D**) The increased apoptosis of BC cells induced by miR-1298 was attenuated with the transfection of ADAM9. *P<0.05, ***P<0.001.

To validate the role of ADAM9 in miR-1298-mediated suppressive function in the progression of BC, the expression of ADAM9 was restored by transfecting Flag-tagged ADAM9 into both MCF7 and MDA-MB-231 cells. The expression of Flag-ADAM9 was confirmed by Western blot with anti-Flag antibody (Figure 6A). CCK-8 assay demonstrated that overexpression of miR-1298 inhibited the proliferation of BC cells, while re-introduction of ADAM9 significantly attenuated the suppressive role of miR-1298 in both MCF7 and MDA-MB-231 cells (Figure 6B,C). Consistently, transfection of ADAM9 also reversed miR-1298-induced apoptosis of BC cells (Figure 6D). These results indicated the critical role of ADAM9 in mediating the suppressive function of miR-1298 in BC.

## Discussion

Increasing evidence has shown that abnormal expression of miRNAs is beneficial to the tumor formation and progression of BC, which can be used as biomarkers for the prediction and prognosis of cancer patients [22,23]. Although remarkable progress has been made in the treatment of BC, those BC patients with distant metastasis usually have poor prognosis. Therefore, the function and potential application of miRNAs in the development or treatment of BC call for deeper investigation. In the present study, decreased level of miR-1298 was found in BC tissues and cell lines. Down-regulation of miR-1298 was significantly correlated with the advanced progression and shorter 5-year survival rate of BC patients. Overexpression of miR-1298 inhibited the malignant phenotypes of BC cells, indicating the tumor-suppressive role of miR-1298 in BC.

miR-1298 has been demonstrated to serve as tumor suppressor in various malignancies [17–20]. A recent study indicates that miR-1298 is down-regulated in NSCLC and predicted poor prognosis of NSCLC patients [20]. Overexpression of miR-1298 inhibits the proliferation, migration and invasion of NSCLC cells. These findings suggested the potential of miR-1298 as a novel biomarker and therapeutic target for NSCLC. Similarly, significantly decreased expression of miR-1298 in bladder cancer represses the malignant behaviors of bladder cancer cells [19]. Lower expression of miR-1298 is associated with lymph node metastasis and TNM stage of gastric cancer, and predicts poor survival of patients [18]. There are several possible mechanisms that contribute to the dysregulation of miR-1298 in BC. The gene locus of miR-1298 may be located at the chromosome that is lost during the BC development. Additionally, aberrant methylation of promoter region decreased the transcription of miR-1298 in BC. Because miRNAs are processed from the pre-matured miRNA, the dysregulation of miR-1298 in BC may also be resulted from the defects of processing, such as the down-regulation of Dicer. In the present study, overexpression of miR-1298 reduced the proliferation, induced apoptosis and cell cycle arrest of BC cells, suggesting the tumor-suppressive function of miR-1298 in BC. Further study might be necessary to fully elucidate the correlation of miR-1298 with the prognosis of BC patients in a large cohort. Because of the heterogeneous in the pathologic and molecular features of BC, using specific cell lines to investigate BC is limited. Further studies with more different types of BC cells and *in vivo* assays are warranted to validate the inhibitory role of miR-1298 in BC.

Accumulating evidence has demonstrated the overexpression of ADAM9 in cancers and promoted the cancer progression [24–28]. It is reported that ADAM9 facilitates the cancer cell survival and metastasis. Additionally, ADAM9 is also found to contribute to the angiogenesis and vascular remodeling of cancer cells [29]. Due to the critical roles of ADAM9 in cancer, ADAM9 is targeted by numerous miRNAs to modulate the tumorigenesis [30–35]. For example, miR-520f inhibits the epithelial to mesenchymal transition by targeting ADAM9 [30]. ADAM9 is negatively regulated by miR-129-5p and suppresses the progression of gastric cancer [36]. Moreover, miR-126 inhibits the metastasis of prostrate cancer cell through targeting ADAM9 [33]. In this study, our results showed that miR-1298 bound the 3′-UTR of ADAM9 and decreased the expression of ADAM9 in BC cells. Consistently, the level of miR-1298 was inversely correlated with that of the ADAM9 in BC tissues. Restoration of ADAM9 significantly reversed the suppressive function of miR-1298 in regulating the malignant phenotypes of BC cells. Our results identified the novel role of miR-1298/ADAM9 pathway in BC. Interestingly, miR-TARP assay has been widely used to identify the targets of miRNA [37]. To provide more evidence for the function of miR-1298, we are now working on the miR-TRAP assay of miR-1298 to find more targets in addition to ADAM9, which may also play important roles in the development of BC.

In conclusions, our findings suggested the tumor-suppressive function of miR-1298 by targeting ADAM9 in BC, which suggested the potential of miR-1298 as a new regulator and therapeutic target for patients with BC.

## Data Availability

All the data presented in the present study are available from the corresponding author upon reasonable request.

## Abbreviations

ADAM9: a disintegrin and metalloproteinase 9 domain
BC: breast cancer
CCK-8: cell counting kit-8
miRNA: microRNA
Mut: mutant
ORF: open reading frame
RT: room temperature
RT-qPCR: reverse transcription-quantitative PCR
UTR: untranslated region
WT: wildtype
