# Expression of Concern: microRNA-1298 inhibits the malignant behaviors of breast cancer cells via targeting ADAM9

**DOI:** 10.1042/BSR-20201215_EOC

**Published:** 2021-04-09

**Keywords:** ADAM9, breast cancer, miR-1298

The Editorial Office has been made aware of potential issues surrounding the scientific validity of this paper, hence has issued an expression of concern to notify readers whilst the Editorial Office investigates.

